# Simultaneous Detection of Four Foodborne Pathogens in Raw Freshwater Fish Using High-Resolution Melting Analysis

**DOI:** 10.3390/foods14183202

**Published:** 2025-09-15

**Authors:** Shan Shan, Xiaoyu Tong, Wenyu Du, Yin Chen, Long Cheng, Fang Yan, Yujie Zhai, Kui Zhao, Haiyan Ni, Xiaomei Sha, Xiaoqing Liu, Chengwei Liu, Shuanglong Wang, Daofeng Liu

**Affiliations:** 1 Nanchang Key Laboratory of Microbial Resources Exploitation & Utilization from Poyang Lake Wetland, College of Life Science, Jiangxi Normal University, Nanchang 330022, China; ncuskshanshan@163.com (S.S.); txy2039175109@163.com (X.T.); 13065158774@163.com (W.D.); chenyin200501011@outlook.com (Y.C.); cl13632023@163.com (L.C.); nihaiyan16@163.com (H.N.); shaxiaomei1987@sina.com (X.S.); 2 Jiangxi Provincial Key Laboratory of Major Epidemics Prevention and Control, Key Laboratory of Nutrition Diet and Health of Jiangxi Provincial Health Commission, Jiangxi Provincial Center for Disease Control and Prevention, 555 East Beijing Road, Nanchang 330029, China; 416500230046@email.ncu.edu.cn (F.Y.); perfectzhai@163.com (Y.Z.); zk17770843514@163.com (K.Z.); liux14@163.com (X.L.); liuchengwei670@sina.com (C.L.); 3 School of Public Health, Jiangxi Medical Collage, Nanchang University, Nanchang 330019, China; 4 Jiangxi Key Laboratory for Mass Spectrometry and Instrumentation, East China University of Technology, Nanchang 330013, China; jiayou1010@163.com

**Keywords:** raw freshwater fish, foodborne pathogens, high-resolution melting (HRM), food safety

## Abstract

Many countries around the world feature raw fish in their cuisine, which is valued for its unique flavor. However, raw fish may be easily contaminated with foodborne pathogens including *Listeria monocytogenes*, *Salmonella*, *Vibrio parahaemolyticus*, and *Staphylococcus aureus*. Herein, a method was established that integrated a multiplex polymerase chain reaction (PCR) and high-resolution melting (HRM) curve assay for the simultaneous detection of these four foodborne pathogens. The target genes of the bacteria were amplified by PCR and subsequently analyzed using HRM. Differentiation was achieved based on the melting temperature (Tm) values of their respective amplicons. The detection limit of the PCR-HRM assay was 0.02–0.1 ng/µL. In addition, the Tm remained nearly constant across various concentrations of genomic DNA derived from the target bacteria. The assay demonstrated perfect specificity (8/8) and a sensitivity of 5/5 for *L. monocytogenes*, 2/2 for *Salmonella*, 3/3 for *V. parahaemolyticus*, and 3/3 for *S. aureus*. No significant interference occurred when genomic DNA from the four target bacteria was co-extracted with DNA from eight non-target strains. Furthermore, the assay offers advantages including operational simplicity, high efficiency, accurate results, reduced detection time, and lower costs, rendering it well-suited for food safety applications in the aquatic products processing industry.

## 1. Introduction

Many countries around the world feature raw fish in their cuisine. From sushi to ceviche to crudo, these dishes possess fresh taste and unique texture, and are rich in nutrition. China has its own ancient tradition of consuming raw fish, stretching back as far as the Zhou dynasty (1046-256 BCE), and this delicacy has become increasingly fashionable over the centuries [1,2]. Nowadays, particularly in the south of China, people have maintained raw-fish-eating traditions. However, consuming raw fish is often associated with human disease [3,4], as it can be easily contaminated with foodborne pathogens, such as *Listeria monocytogenes* (*L. monocytogenes*), *Salmonella* spp., *Vibrio parahaemolyticus* (*V. parahaemolyticus*), and *Staphylococcus aureus* (*S. aureus*) [4,5].

*L. monocytogenes* is a zoonotic pathogen that can easily infect the elderly, pregnant women, infants, and other immunocompromised individuals. It causes symptoms like fever and muscle pain, and can lead to severe septicemia and meningitis, with a fatality rate as high as 30% [6,7,8]. *Salmonella* infection usually results in symptoms such as fever, abdominal pain, diarrhea, nausea, and sometimes vomiting [9]. The onset of disease symptoms occurs 6–72 h (usually 12–36 h) after ingestion of *Salmonella*, and illness lasts 2–7 days. After being infected with *V. parahaemolyticus*, patients may exhibit symptoms such as abdominal tenderness, diarrhea, fever, and vomiting [10]. In severe cases, it can also lead to sepsis, shock, and death [11]. Although death caused by *S. aureus* is relatively rare, the infection inflicts significant damage to the intestinal tract, and the resulting poisoning reaction can be more severe [12]. These foodborne pathogens are widely distributed not only in aquatic products but also in the surrounding environment, such as water bodies, fishing gear, and processing equipment, leading to serious contamination. Therefore, establishing high-throughput detection methods that are sensitive, rapid, and accurate is of paramount importance for ensuring food safety.

Many efforts have been devoted to developing various assays for efficiently detecting bacteria, but most methods may only be capable of detecting a single species of bacteria [13]. Therefore, developing an accurate and fast multiplex detection technology is especially useful in cases where there may be multiple types of bacteria present in a single sample. The current methods for detecting foodborne pathogens mainly include traditional culture methods, immunological methods, and molecular biological methods [14]. Traditional culture methods are typically used for testing one species of bacteria, which are time-consuming and labor-intensive [15]. Immunological methods are based on the specific reaction between antigens and antibodies, such as ELISA, lateral flow strips, and biosensors. Lv et al. [16] developed a multicolor and ultrasensitive ELISA platform for detecting *Escherichia coli* O157:H7, *Salmonella*, and *L. monocytogenes* simultaneously with three different fluorescences. Most immunoassays are relatively expensive and prone to false-positive results [17]. Molecular microbiology methods are increasingly used for bacterial detection, offering faster, more accurate, and more precise results, and are being applied across a wide range of settings [18,19]. In terms of multiplex detection, multiple polymerase chain reaction (mPCR) seems to be effective for the simultaneous detection of numerous pathogenic bacteria in practical applications [20]. However, the dependence of multiplex isothermal amplification and PCR methods on fluorescent probes makes these detection assays very expensive, which limits the application of these detection methods. Additionally, electrophoresis and lateral flow strip-based multiplex test lines could also be used to identify the amplicons [21]. But for all these methods, the reaction tubes need to be opened, which can easily cause aerosol pollution [22].

The main objective of this study was to detect the four most common pathogenic bacteria (including *L. monocytogenes*, *Salmonella*, *V. parahaemolyticus*, and *S. aureus*) in a single tube without causing aerosol pollution. The target genes of the bacteria could be amplified by PCR and saturated dye stained the double-stranded DNA (dsDNA). Then the reaction temperature gradually increased, causing the amplicons to melt. The melting temperature (Tm) of the amplicons was obtained during HRM assay, then the four pathogenic bacteria could be confirmed according to their specific Tm. This method has the advantages of being low cost and rapid, and could be used for detecting the four foodborne pathogens in a single sample.

## 2. Materials and Methods

### 2.1. Bacteria Strain Culture and DNA Extraction

In this study, all bacteria used were from Jiangxi Provincial Centre for Disease Control and Prevention (Jiangxi CDC). The strains were listed in the online Supplemental Materials (Table S1). The bacteria cells were cultured in 10 mL tryptic soy broth (TSB, Beijing Land Bridge Technology Co., Ltd., Beijing, China) and incubated in a shaking incubator at 37 °C and 180 rpm for 18 h. Then, 1 mL bacteria solution was centrifuged (8000 rpm, 10 min) and the bacteria cells were obtained. The precipitate was washed with ultrapure water once, and 200 μL ultrapure water was added to dissolve the bacteria cells. The bacteria solution was heated in a heat block at 100 °C for 10 min and then put in the fridge at 4 °C for 5 min. After that the solution was centrifuged at 12,000 rpm for 5 min, and the supernatant was used as the DNA template [23]. The DNA concentration was determined by Nanodrop Lite Spectrophotometer (Thermo Fisher Scientific, Wilmington, DE, USA). The final DNA concentration of the four pathogens was adjusted to 50 ng/µL. The ultrapure water used in this experiment comes from Synergy ultrapure water system (Merck KGaA, Darmstadt, Germany).

### 2.2. Primer Design and Synthesis

Primer sets of the *L. monocytogenes* virulence gene *hlyA*, *Salmonella* virulence gene *invA*, *V. parahaemolyticus* virulence gene *tlh*, and *S. aureus* virulence gene *nuc* were designed for the specific PCR assay [24,25,26,27]. The gene sequences of *hlyA*, *invA*, *tlh*, and *nuc* were retrieved from NCBI GenBank database (https://www.ncbi.nlm.nih.gov/nuccore/, accessed on 24 December 2024, MD, USA). The most suitable primer sequences were designed by Primer Premier 5.0 software (PREMIER Biosoft, Canada) and are shown in Table S2.

### 2.3. Establishment of Singleplex PCR-HRM Method

The singleplex PCR-HRM assay was performed to determine the Tm of the specific amplicons. A total of 20 µL of the following reaction mixture was prepared in a micro-centrifuge tube: 10 µL of MeltDoctor™ HRM Master Mix (Applied Biosystems™, Waltham, MA, USA), 2 µL of primer mixture (the concentrations of all upstream primers and downstream primers at a final concentration of 6 µM each), 2 µL of bacteria DNA as the template, and 7 µL of ultrapure water. Ultrapure water was used instead of the template in the negative control. Three parallel experiments were carried out. The Applied Biosystems 7500 Fast Real-Time PCR Instrument (Thermo Fisher Scientific, Waltham, MA, USA) was used for the PCR-HRM assay. The following conditions were applied for the PCR: pre-denaturation at 95 °C for 10 min, followed by 25 cycles of denaturation at 95 °C for 15 s and amplification at 60 °C for 1 min. The HRM assay was performed following PCR: pre-denaturation at 95 °C for 15 s and annealing at 60 °C for 1 min, from 60 °C to 90 °C at a rate of 0.04 °C/s.

### 2.4. Establishment of Quadruple PCR-HRM Assay

A total of 20 µL of the following reaction mixture was prepared in a tube: 10 µL of MeltDoctor™ HRM Master Mix, 3 µL of primer mixture, 2 µL of bacteria DNA as the template, and 6 µL of ultrapure water. The ratios of *hlyA*, *nuc*, *tlh*, and *invA* were set at 1:1:1:1, 4:3:2:1, and 6:2:2:1. The final concentration of the *invA* primer remained at 0.1 µM, while the concentrations of all other primers were adjusted based on their ratio to *invA*. The PCR-HRM assay was performed using the same procedures described in Section 2.3. The amplification products were subjected to capillary electrophoresis (Qiagen GmbH, Hilden, Germany). The parameters used for capillary electrophoresis are as follows: the cartridge type is DNA High Resolution, the process profile is “*NewProcesPprofile”, the method is OM800 in which the sample injection voltage is 5 kV, the sample injection time is 20 s, the separation voltage is 3 kV, the separation time is 800 s, the number of runs per row is 7 rounds, the alignment makers are QX 15 bp-3 kb, and the amount of sample added is 15 μL.

### 2.5. Detection Limit of Quadruple PCR-HRM Assay

The concentration sof DNA samples obtained from *L. monocytogenes*, *S. typhimurium*, *V. parahaemolyticus*, and *S. aureus* were diluted to different concentration gradients (50 ng/µL, 25 ng/µL, 5 ng/µL, 1 ng/µL, 0.5 ng/µL, 0.1 ng/µL, and 0.02 ng/µL) as the templates. The PCR-HRM assay followed the protocols outlined in Section 2.3. Additionally, ultrapure water was used as a blank control, and the assays were performed in triplicate.

### 2.6. Sensitivity, Specificity, and Anti-Interference Assay

Bacterial target genes were amplified by PCR, and the amplicon Tm values were determined during HRM analysis. The resulting Tm peak served as a distinct signature for identifying target strains within the sample. DNA samples extracted from five strains of *L. monocytogenes*, two strains of *S. typhimurium*, three strains of *V. parahaemolyticus*, and three strains of *S. aureus* were detected by quadruple PCR-HRM assay for evaluating the sensitivity of the current assay. In addition, the specificity of the quadruple PCR-HRM assay was assessed by detecting other pathogenic bacteria, including enteropathogenic *Escherichia coli* (EPEC), enteroinvasive *Escherichia coli* (EIEC), enterotoxigenic *Escherichia coli* (ETEC), Shiga toxin-producing *Escherichia coli* (STEC), and enteroaggregative *Escherichia coli* (EAEC), *Shigella*, *Enterobacter sakazakii*, and *Staphylococcus argenteus*. The PCR-HRM assay conducted the protocols outlined in Section 2.3.

In addition, the anti-interference assay proceeded to evaluate the applicability of quadruple PCR-HRM assay. The DNA mixture including *L. monocytogenes*, *S. typhimurium*, *V. parahaemolyticus*, *S. aureus*, EPEC, EIEC, ETEC, STEC, EAEC, *Shigella*, and *Enterobacter sakazakii* was detected by PCR-HRM assay following the protocols outlined in Section 2.3.

### 2.7. Detection and Subtyping of Four Pathogenic Bacteria in Sliced Raw Freshwater Fish

Grass carp were purchased from the local supermarket (Rainbow supermarket, Nanchang, China), sectioned into thin slices, and sterilized with ultraviolet for 3 h. For spiked sample preparation, 1 mL of *L. monocytogenes*, *S. typhimurium*, *V. parahaemolyticus*, and *S. aureus* (100 CFU/mL) was added to 25 g portions of fish sample, respectively. And 1 mL of ultrapure water was added to the fish sample as a control group. Subsequently, each sample was transferred to 225 mL of 3% (*w*/*v*) NaCl-supplemented Alkaline Peptone Water (APW) medium and incubated with continuous shaking at 180 rpm for 10 h. Then, 1 mL of the mixture was transferred to a tube and the DNA was extracted according to Section 2.1 and then detected by the PCR-HRM assay.

### 2.8. Statistical Analysis

Data were expressed as mean values ± standard deviation (SD), and Origin 2024b software was used for graphing.

## 3. Results

### 3.1. Singleplex PCR-HRM Assay

One of the most effective ways to confirm the presence of a target bacterium is by detecting its virulence factors, such as listeriolysin O (LLO) in *L. monocytogenes*. The LLO-encoding gene (*hlyA*) is found only in virulent strains of this species and is essential for virulence [28]. The *invA* gene is a common specific target for *Salmonella* detection methods and has a highly conserved sequence related to virulence and invasion [25]. The gene encoding the thermostable hemolysin (*tlh*) is a signature molecular marker for *V. parahaemolyticus* [10]. The *nuc* gene encodes thermally stable nuclease and is widely used as a specific target for detecting *S. aureus* [29]. Therefore, *hlyA*, *nuc*, *tlh*, and *invA* were chosen as the target genes to detect *L. monocytogenes*, *S. aureus*, *V. parahaemolyticus*, and *Salmonella*, respectively.

**Figure 1:**
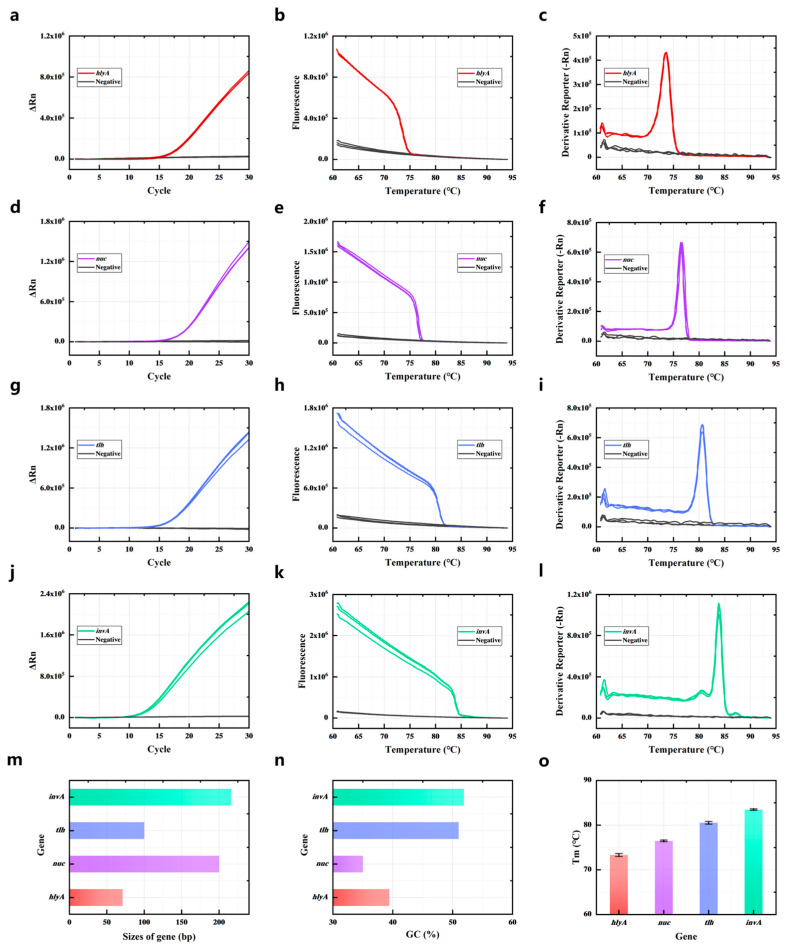
Single melting curve identification of four common pathogenic bacteria. PCR amplification curves of *hlyA* (**a**), *nuc* (**d**), *tlh* (**g**), and *invA* (**j**); PCR amplicon melting curves of *hlyA* (**b**), *nuc* (**e**), tlh (**h**), and *invA* (**k**); HRM curve of *hlyA* (**c**), *nuc* (**f**), *tlh* (**i**), and *invA* (**l**). (**m**) The sizes of the four target gene PCR amplicons. (**n**) The GC contents of the four target gene PCR amplicons. (**o**) The Tm of the four target gene PCR amplicons. All assays were performed in triplicate.

Firstly, the target genes of the bacteria were amplified by singleplex PCR, and the saturated dye stained the amplicons that were double-stranded DNA (dsDNA). Figure 1a,d,g,j show that the target gene of the bacteria could be amplified by the primers (Table S2), and as PCR proceeded, the fluorescence increased because the fluorescent dye (Sybr Green I) was binding with the amplicons. Then the reaction temperature gradually increased, causing the amplicons to melt. DNA denaturation (melting) is the process of separating dsDNA into two single strands. When the dsDNA melted into single strands, the dye was released, causing a change in fluorescence; the fluorescence decreased, as shown in Figure 1b,e,h,k. When the temperature reached the Tm value of dsDNA, there was a sharp drop in the fluorescence intensity. A melt curve could be obtained by plotting the negative derivative of fluorescence versus (-dF/dT). As shown in Figure 1c,f,i,l, the amplification products of *hlyA*, *nuc*, *tlh*, and *invA* exhibited distinct melting temperatures. The singleplex PCR-HRM assay was performed in triplicate, determining that the average values of Tm of *hlyA*, *nuc*, *tlh*, and *invA* amplicons were 73.626 ± 0.325 °C, 76.614 ± 0.169 °C, 80.358 ± 0.290 °C, and 83.774 ± 0.173 °C (Figure 1c,f,i,l). The Tm values of the four gene amplicons had clear differences; these primers were suitable for use in the multiplex PCR assay.

The Tm of the amplification products is determined by the GC content, length, and sequence of the dsDNA [30]. Therefore, the primers of target genes (*hlyA*, *nuc*, *tlh*, and *invA*) were designed and chosen according to the length and GC content of their amplification products. As shown in Figure 1m, the length of *hlyA* amplicons was 71 bp, the length of *nuc* amplicons was 200 bp, the length of *tlh* amplicons was 100 bp, and the length of *invA* amplicons was 216 bp. The GC content of *hlyA* amplicons was 39.4%, and the GC content of *nuc* amplicons was 35%. The GC content of *tlh* amplicons was 51% and the GC content of *invA* amplicons was 51.9% (Figure 1n). The longer length and higher GC content of the dsDNA meant it could obtain a higher Tm. Thus, the *invA* amplicon had longest length and highest GC content of the four target gene amplicons. So the Tm of the *invA* amplicon was the highest at 83.774 ± 0.173 °C (Figure 1o). The Tm of the *hlyA* amplicon was the lowest because it had a short length and low GC content. Although the *nuc* amplicon had the lowest GC content of the four gene amplicons, the length of the *nuc* amplicon was 200 bp, which was much longer than the *hlyA* length of 71 bp. The Tm of the *nuc* amplicon (76.614 ± 0.169 °C) was higher than the *hlyA* amplicon (73.626 ± 0.325 °C). The length of *tlh* (100 bp) was shorter than *invA* (216 bp) and *nuc* (200 bp). The GC content of *tlh* (51%) was almost the same as *invA* (51.9%) and much higher than the *nuc* amplicon (39.4%). The Tm of the *tlh* amplicon (80.358 ± 0.290 °C) was higher than the *nuc* amplicon (76.614 ± 0.169 °C).

### 3.2. Quadruplex PCR-HRM Analysis

**Figure 2:**
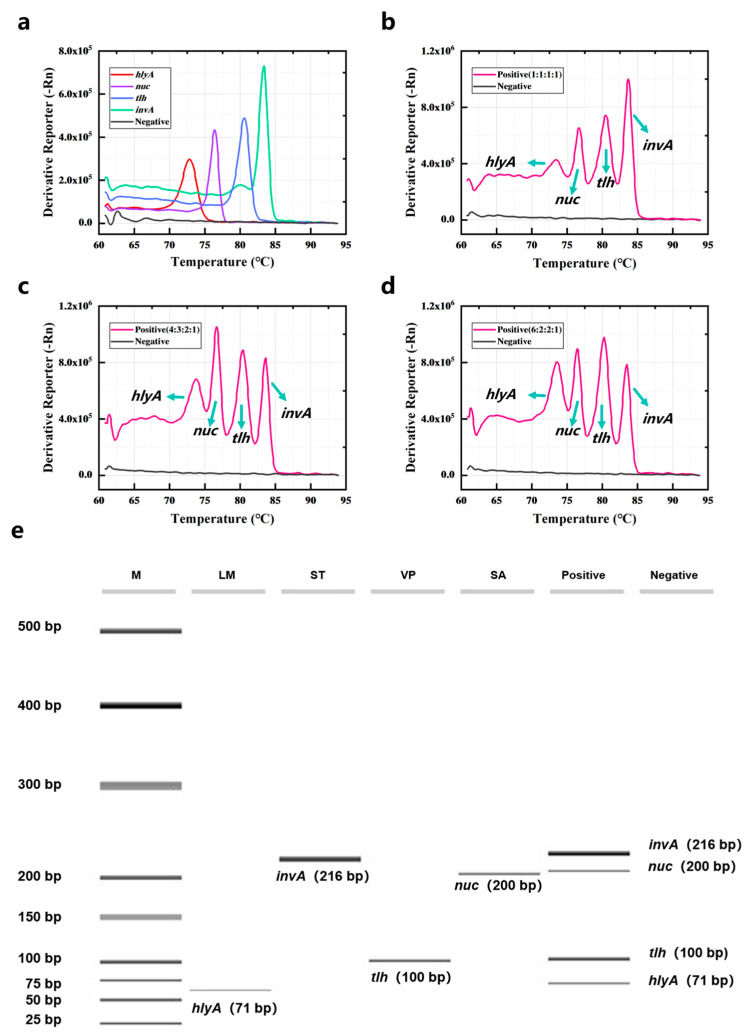
Quadruple PCR combined with HRM assay for the detection of four common pathogenic bacteria. (**a**) The four bacteria were detected by PCR-HRM assay. The four bacteria mixtures were detected by PCR-HRM assay when the primer concentration ratios of *hlyA*–*nuc*–*tlh*–*invA* were (**b**) 1:1:1:1, (**c**) 4:3:2:1, (**d**) 6:2:2:1. (**e**) The capillary electrophoresis of the products was amplified by the quadruple PCR-HRM assay. M was marker and contained different DNA from 25 bp to 500 bp.

The DNA of *L. monocytogenes*, *S. aureus*, *V. parahaemolyticus*, and *S. typhimurium* was detected by quadruple PCR-HRM assay. Results are shown in Figure 2a; the four bacteria could be detected by the current method, and the peak positions of the melting curves of the four specific amplicons do not overlap. A DNA mixture containing all four bacteria was also analyzed via quadruplex PCR-HRM assay. Figure 2b revealed that the Derivative Reporter (-Rn) values for *hlyA*, *nuc*, and *tlh* were lower than those for *invA*, indicating lower amplification efficiency of the former primers. To improve the amplification efficiency, primer concentrations for *hlyA*, *nuc*, and *tlh* were increased (Figure 2c,d). When the primer concentration ratio of *hlyA*–*nuc*–*tlh*–*invA* was 6:2:2:1, a melt curve with four distinct sets of peaks was obtained (Figure 2d). Furthermore, varying primer concentration ratios did not affect the Tm values of the four gene amplicons. The Tm values determined by quadruplex PCR-HRM assay was almost the same as those detected by singleplex PCR-HRM assay. Specifically, the Tm value for *L. monocytogenes* was 73.4 ± 0.325 °C, for *S. aureus* it was 76.65 ± 0.169 °C, *V. parahaemolyticus* was 80.512 ± 0.290 °C, and *S. typhimurium* was 83.687 ± 0.173 °C. Amplification products generated by quadruplex PCR-HRM assay were subjected to capillary electrophoresis. Results are shown in Figure 2e, confirming amplicons sizes of 71 bp (*hlyA*), 216 bp (*invA*), 100 bp (*tlh*), and 200 bp (*nuc*). Four distinct bands corresponding to these fragment sizes were observed when detecting all four bacteria simultaneously. The negative control lane had no band, and all lanes were clear, indicating that the specificity of multiplex PCR amplification for gene fragments is good.

### 3.3. Detection Limit of PCR-HRM Assay

**Figure 3:**
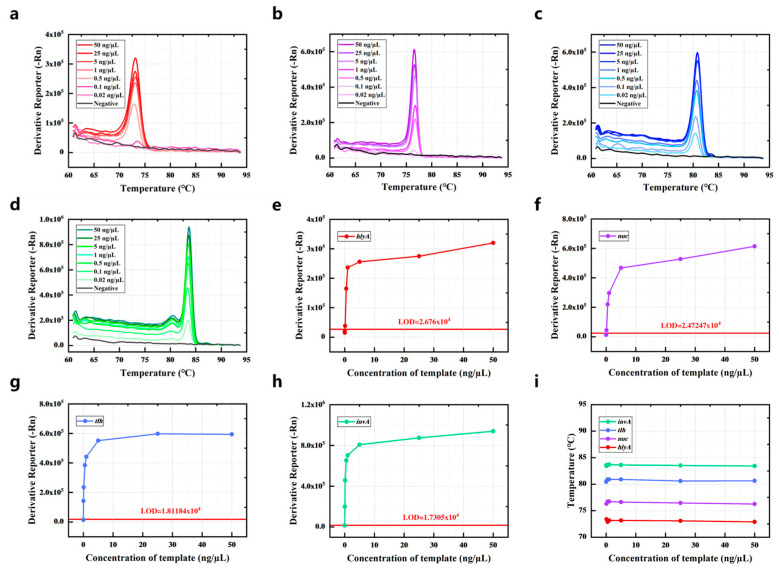
The detection limit of the quadruple PCR-HRM system. The HRM curve of different concentrations of genomic DNA was obtained from (**a**) *L. monocytogenes*, (**b**) *S. typhimurium*, (**c**) *V. parahaemolyticus*, and (**d**) *S. aureus*. The Derivative Reporter (-Rn) of different concentrations of genomic DNA was obtained from (**e**) *L. monocytogenes*, (**f**) *S. typhimurium*, (**g**) *V. parahaemolyticus*, and (**h**) *S. aureus* at their Tm. (**i**) The influence of the concentration of genomic DNA on the Tm.

The detection limit of the current method was determined by detecting the dilutions of genomic DNA obtained from the strains. The genomic DNA of the strains spanning concentrations from 0.02 to 50 ng/μL was detected by PCR-HRM assay, with corresponding HRM curves displayed in Figure 3a–d. The proposed method had a detection limit of 0.1 ng/μL for *L. monocytogenes* and *S. aureus*, while the detection limit of *Salmonella* and *V. parahaemolyticus* was 0.02 ng/μL (Figure 3e–h). Due to varying amplification efficiencies among primer pairs, the limit of detection ranges from 0.02 to 0.1 ng/μL for detecting different pathogens. Notably, the Tm value remained nearly constant when measuring genomic DNA across a wide concentration range (Figure 3i). Therefore, the concentration of strains has minimal impact on results within a certain range.

### 3.4. Sensitivity, Specificity, and Anti-Interference Ability of PCR-HRM Assay

**Figure 4:**
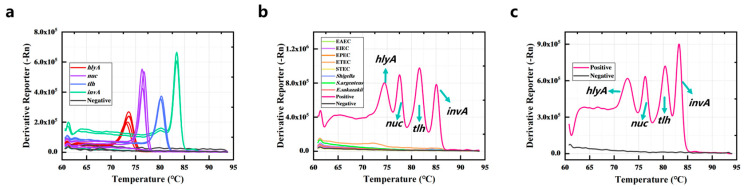
Sensitivity (**a**), specificity (**b**), and anti-interference ability (**c**) of the PCR-HRM assay.

The sensitivity analysis was performed using some clinical strains from Jiangxi CDC, comprising five strains of *L. monocytogenes*, two strains of *Salmonella*, three strains of *V. parahaemolyticus*, and three strains of *S. aureus*. The results of the sensitivity analysis are shown in Figure 4a; the DNA extracted from the target bacteria could be detected accurately by the PCR-HRM assay. The average value of Tm of *hlyA*-specific amplicons was 73.4 ± 0.325 °C, *nuc*-specific amplicons was 76.65 ± 0.169 °C, *tlh*-specific amplicons was 80.512 ± 0.290 °C, and *invA*-specific amplicons was 83.687 ± 0.173 °C.

Eight standard strains including EPEC, EIEC, ETEC, STEC, EAEC, *Shigella*, *Enterobacter sakazakii*, and *Staphylococcus argenteus* were used to evaluate the specificity of the PCR-HRM assay. Figure 4b demonstrates that these strains and the negative control did not have amplification. The results show that the specificity of the PCR-HRM assay was good. In addition, for evaluating the anti-interference ability of the current method, the genomic DNA of the above eight non-target strains was mixed with the genomic DNA of the four target bacteria, and the DNA mixture was detected by PCR-HRM assay. Figure 4c shows that the four target genes were accurately detected with unchanged Tm values, despite the presence of non-target DNA.

### 3.5. Detection of the Four Foodborne Pathogens in Sliced Raw Freshwater Fish

**Figure 5:**
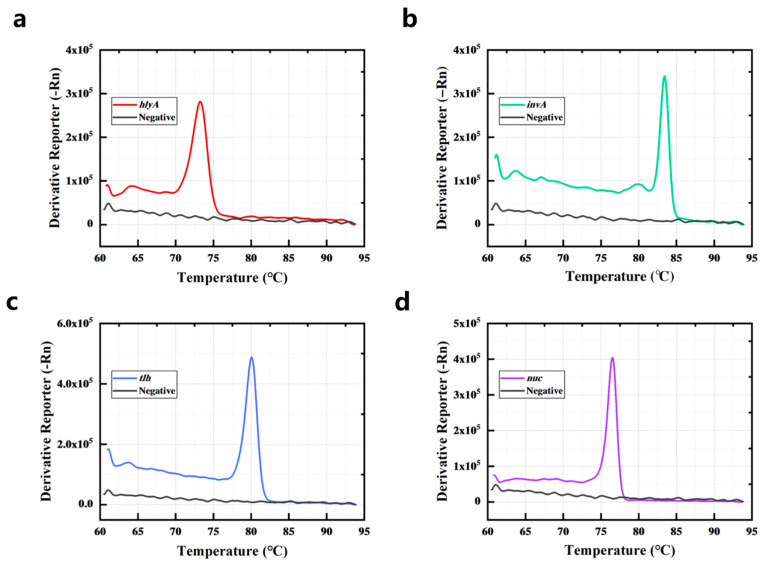
*L. monocytogenes* (**a**), *S. typhimurium* (**b**), *V. parahaemolyticus* (**c**), and *S. aureus* (**d**) in sliced raw freshwater fish were detected by the PCR-HRM assay.

To further evaluate the detection efficiency of the PCR-HRM assay in the real sample, the four bacteria (*L. monocytogenes*, *S. typhimurium*, *V. parahaemolyticus*, and *S. aureus*) were spiked in sliced raw freshwater fish. As shown in Figure 5a–d, *L. monocytogenes* has a melting curve peak at 73.239 °C (*hlyA*), *S. aureus* has a melting curve peak at 76.483 °C (*nuc*), *V. parahaemolyticus* has a melting curve peak at 80.095 °C (*tlh*), and *S. typhimurium* has a melting curve peak at 83.411 °C (*invA*). Matrix interference from actual fish specimens did not significantly affect pathogen detection outcomes.

## 4. Discussion

Many countries around the world boast raw fish in their cuisine due to these dishes’ own fresh taste, unique texture, and richness in nutrition. However, raw fish may be easily contaminated with foodborne pathogens including *L. monocytogenes*, *Salmonella*, *V. parahaemolyticus*, and *S. aureus*. Therefore, establishing high-throughput detection methods that are sensitive, rapid, and accurate is of paramount importance for ensuring food safety. While PCR enables robust, simultaneous detection of numerous pathogenic bacteria in practical settings [20], its utility is often limited by cost. Like multiplex isothermal amplification, PCR depends on expensive fluorescent probes, hindering its accessibility. Although alternative readouts via electrophoresis or multi-line lateral flow strips offer potential solutions [21], these approaches share a significant limitation: the requirement to open reaction tubes, which carries a substantial risk of aerosol contamination [22]. HRM analysis is a post-PCR technique applied to double-stranded DNA. It is renowned for its exceptional sensitivity in detecting single-base alterations; HRM effectively identifies genetic mutations and single nucleotide polymorphisms (SNPs) within amplified targets [31,32]. Owing to its simplicity, cost-effectiveness, and ease of use, HRM has seen widespread adoption in both clinical diagnostics [33] and food safety testing [34,35]. Notably, Harrison and Hanson [36] employed HRM for the rapid detection of sequence type 131 *E. coli*, a globally significant multidrug-resistant clone. Furthermore, both researchers and our group have successfully adapted HRM curve analysis to detect various pathogenic *E. coli* strains [6,37,38]. Herein, a PCR-HRM assay method for the simultaneous detection of *L. monocytogenes*, *S. typhimurium*, *V. parahaemolyticus*, and *S. aureus* in aquatic products was established.

The primers of the target genes were designed, and their corresponding amplicons’ Tm values were theoretically calculated using the DINAMelt server (results listed in Table S3). Subsequently, each of the four bacteria was detected via singleplex PCR. Both the PCR and HRM analysis processes were recorded and analyzed, with results presented in Figure 1. The identity of all PCR products was confirmed by comparing experimental Tm values to theoretical predictions from the DINAMelt server, as they exhibited consistent trends. Since the Tm of the amplification products depended on the GC content, length, and sequence of the duplex [34], the distinct Tm values observed among the four gene amplicons indicated that the primers could be used for the multiplex PCR assay. The disparity in amplification efficiencies among primer pairs necessitated optimizing the *hlyA*–*nuc*–*tlh*–*invA* primer concentration ratio to 6:2:2:1, which resulted in a melting curve characterized by four discrete peaks. The detection limits were 0.1 ng/µL for *hlyA* and *nuc* and 0.02 ng/µL for *invA* and *tlh*. Furthermore, Tm values remained stable across varying concentrations of genomic DNA from the target bacteria. To validate performance in complex matrices, the four bacteria were spiked into sliced raw freshwater fish and detected using the PCR-HRM assay. These results demonstrated that the method could detect target bacteria without influence from the food matrix or non-target bacteria.

## 5. Conclusions

In conclusion, we established a PCR-HRM assay method for the simultaneous detection of *L. monocytogenes*, *S. typhimurium*, *V. parahaemolyticus*, and *S. aureus* in aquatic products. The primer concentration ratio of *hlyA*–*nuc*–*tlh*–*invA* was 6:2:2:1, and a melt curve with four distinct peaks was obtained. The detection limit was 0.1 ng/µL for *hlyA* and *nuc* and 0.02 ng/µL for *invA* and *tlh*. In addition, the Tm remained stable across varying concentrations of genomic DNA from target bacteria. The specificity of this assay was 8/8, and the sensitivity of the assay was 5/5 for *L. monocytogenes*, 2/2 for *Salmonella*, 3/3 for *V. parahaemolyticus*, and 3/3 for *S. aureus*. Crucially, results were unaffected when the genomic DNA from the four target bacteria was mixed with that of eight non-target strains. Compared to conventional multiplex PCR–electrophoresis, our method significantly reduces analysis time, streamlines the workflow, and increases efficiency, conferring substantial practical utility, particularly for early detection, definitive diagnosis, and rapid intervention in foodborne diarrheal diseases.

## Data Availability

The original contributions presented in this study are included in the article/Supplementary Material. Further inquiries can be directed to the corresponding author.

## Supplementary Materials

The following supporting information can be downloaded at: https://www.mdpi.com/article/10.3390/foods14183202/s1, Table S1: List of the strains utilized and their sources; Table S2: Primer sequences; Table S3: Theoretical Tm prediction of amplification products for different gene types.

## References

[1] Zhang H., Sun C., Wang Z., Che B. (2021). Seafood Consumption patterns and affecting factors in urban China: A field survey from six cities. Aquac. Rep..

[2] Smith H.A. (2022). Food, health, and nutrition in Chinese history. Hist. Compass.

[3] Li N., Wu X., Zhuang W., Xia L., Chen Y., Wu C., Rao Z., Du L., Zhao R., Yi M. (2020). Fish consumption and multiple health outcomes: Umbrella review. Trends Food Sci. Technol..

[4] Novoslavskij A., Terentjeva M., Eizenberga I., Valciņa O., Bartkevičs V., Bērziņš A. (2016). Major foodborne pathogens in fish and fish products: A review. Ann. Microbiol..

[5] Li Y., Pei X., Yan J., Liu D., Zhang H., Yu B., Li N., Yang D. (2019). Prevalence of foodborne pathogens isolated from retail freshwater fish and shellfish in China. Food Control.

[6] Liu Y., Ceruso M., Gunther N., Pepe T., Cortesi M., Fratamico P. (2012). Construction of *Listeria monocytogenes* mutants with in-frame deletions in putative ATP-binding cassette (ABC) transporters and analysis of their growth under stress conditions. J. Microb. Biochem. Technol..

[7] Quereda J.J., Morón-García A., Palacios-Gorba C., Dessaux C., García-del Portillo F., Pucciarelli M.G., Ortega A.D. (2021). Pathogenicity and virulence of *Listeria monocytogenes*: A trip from environmental to medical microbiology. Virulence.

[8] Radoshevich L., Cossart P. (2018). *Listeria monocytogenes*: Towards a complete picture of its physiology and pathogenesis. Nat. Rev. Microbiol..

[9] Galán-Relaño Á., Valero Díaz A., Huerta Lorenzo B., Gómez-Gascón L., Mena Rodríguez M.Á., Carrasco Jiménez E., Pérez Rodríguez F., Astorga Márquez R.J. (2023). *Salmonella* and salmonellosis: An update on public health implications and control Strategies. Animals.

[10] Li L., Meng H., Gu D., Li Y., Jia M. (2019). Molecular mechanisms of *Vibrio parahaemolyticus* pathogenesis. Microbiol. Res..

[11] Wang R., Zhong Y., Gu X., Yuan J., Saeed A.F., Wang S. (2015). The pathogenesis, detection, and prevention of *Vibrio parahaemolyticus*. Front. Microbiol..

[12] Salgado-Pabón W., Tran P.M., Morris J.G., Vugia D.J. (2021). Chapter 25–Staphylococcal Food Poisoning. Foodborne Infections and Intoxications.

[13] Song X., Li W., Wu L., Lv T., Zhang Y., Sun J., Shentu X., Yu X., Wu Y. (2023). Detection of *Vibrio parahaemolyticus* based on magnetic and upconversion nanoparticles combined with aptamers. Foods.

[14] Mazur F., Tjandra A.D., Zhou Y., Gao Y., Chandrawati R. (2023). Paper-based sensors for bacteria detection. Nat. Rev. Bioeng..

[15] Kabiraz M.P., Majumdar P.R., Mahmud M.M.C., Bhowmik S., Ali A. (2023). Conventional and advanced detection techniques of foodborne pathogens: A comprehensive review. Heliyon.

[16] Lv X., Huang Y., Liu D., Liu C., Shan S., Li G., Duan M., Lai W. (2019). Multicolor and ultrasensitive enzyme-linked immunosorbent assay based on the fluorescence hybrid chain reaction for simultaneous detection of pathogens. J. Agric. Food Chem..

[17] Liu Q., Jin X., Cheng J., Zhou H., Zhang Y., Dai Y. (2023). Advances in the application of molecular diagnostic techniques for the detection of infectious disease pathogens (Review). Mol. Med. Rep..

[18] Garg N., Ahmad F.J., Kar S. (2022). Recent advances in loop-mediated isothermal amplification (LAMP) for rapid and efficient detection of pathogens. Curr. Res. Microb. Sci..

[19] Luo Y., Shan S., Wang S., Li J., Liu D., Lai W. (2022). Accurate Detection of Salmonella Based on Microfluidic Chip to Avoid Aerosol Contamination. Foods.

[20] Tombuloglu H., Sabit H., Al-Khallaf H., Kabanja J.H., Alsaeed M., Al-Saleh N., Al-Suhaimi E. (2022). Multiplex real-time RT-PCR method for the diagnosis of SARS-CoV-2 by targeting viral N, RdRP and human RP genes. Sci. Rep..

[21] Shan S., Huang Y., Huang Z., Long Z., Liu C., Zhao X., Xing K., Xiao X., Liu J., Huang Y. (2021). Detection of stx1 and stx2 and subtyping of Shiga toxin-producing *Escherichia coli* using asymmetric PCR combined with lateral flow immunoassay. Food Control.

[22] Starolis M.W. (2024). The contamination monitoring toolbox: Best practices for molecular microbiology testing. Clin. Microbiol. Newsl..

[23] Lee S.-Y., Kim U., Kim Y., Lee S.J., Park E.Y., Oh S.-W. (2023). Enhanced detection of *Listeria monocytogenes* using tetraethylenepentamine-functionalized magnetic nanoparticles and LAMP-CRISPR/Cas12a-based biosensor. Anal. Chim. Acta.

[24] Shi D., Shi H. (2022). Combining loop-mediated isothermal amplification and nanozyme-strip for ultrasensitive and rapid detection of viable *Listeria monocytogenes* cells and biofilms. LWT.

[25] Buehler A.J., Wiedmann M., Kassaify Z., Cheng R.A. (2019). Evaluation of *invA* diversity among *Salmonella* species suggests why some commercially available rapid detection kits may fail to detect multiple *Salmonella* subspecies and species. J. Food Prot..

[26] Park S.B., Zhang Y. (2024). Innovative multiplex PCR assay for detection of *tlh*, *trh*, and tdh denes in *Vibrio parahaemolyticus* with reference to the U.S. FDA’s bacteriological analytical manual (BAM). Pathogens.

[27] Karimzadeh R., Ghassab R.K. (2022). Identification of nuc nuclease and sea enterotoxin genes in *Staphylococcus aureus* isolates from nasal mucosa of burn hospital staff: A cross-sectional study. New Microbes New Infect..

[28] Churchill R.L.T., Lee H., Hall J.C. (2006). Detection of *Listeria monocytogenes* and the toxin listeriolysin O in Food. J. Microbiol. Methods.

[29] Ibraheim H., Fayez R., Jasim A., Gharban H. (2023). Role of nuc gene in *Staphylococcus aureus* to phagocytic activity in different cattle infections. Open Veter- J..

[30] Wittwer C.T., Hemmert A.C., Kent J.O., Rejali N.A. (2024). DNA melting analysis. Mol. Asp. Med..

[31] Narimisa N., Amraei F., Sholeh M., Mirkalantari S., Razavi S., Kalani B.S., Lotfollahi L., Jazi F.M. (2022). Genotyping of Listeria monocytogenes isolates by high-resolution melting curve (HRM) analysis of tandem repeat locus. Braz. J. Infect. Dis..

[32] Guion C.E., Ochoa T.J., Walker C.M., Barletta F., Cleary T.G. (2008). Detection of Diarrheagenic *Escherichia coli* by use of melting-curve analysis and real-time multiplex PCR. J. Clin. Microbiol..

[33] Dehbashi S., Tahmasebi H., Sedighi P., Davarian F., Arabestani M.R. (2020). Development of high-resolution melting curve analysis in rapid detection of *vanA* gene, *Enterococcus faecalis*, and *Enterococcus faecium* from clinical isolates. Trop. Med. Health.

[34] Yan D., Ma Y., Wang H., Jia W., Niu X., Wang H., Zou W., Wang L. (2025). High ionic conductivity conjugated artificial solid electrolyte interphase enabling stable lithium metal batteries. Green Chem..

[35] Forghani F., Singh P., Seo K.-H., Oh D.-H. (2016). A novel pentaplex real time (RT)- PCR high resolution melt curve assay for simultaneous detection of emetic and enterotoxin producing *Bacillus cereus* in food. Food Control.

[36] Harrison L.B., Hanson N.D. (2017). High-Resolution Melting Analysis for Rapid Detection of Sequence Type 131 *Escherichia coli*. Antimicrob. Agents Chemother..

[37] Velez F.J., Bosilevac J.M., Delannoy S., Fach P., Nagpal R., Singh P. (2022). Development and validation of high-resolution melting assays for the detection of potentially virulent strains of *Escherichia coli* O103 and O121. Food Control.

[38] Singh P., Cubillos G., Kirshteyn G., Bosilevac J.M. (2020). High-resolution melting real-time PCR assays for detection of *Escherichia coli* O26 and O111 strains possessing shiga toxin genes. LWT.

