# Supplementary material for: Simultaneous Detection of Four Foodborne Pathogens in Raw Freshwater Fish Using High-Resolution Melting Analysis
Source: Foods. 2025 Sep 15;14(18):3202. doi: 10.3390/foods14183202 (PMC12469668; doi:10.3390/foods14183202)
Supplement: Supplementary file 1 [file foods-14-03202-s001.zip › foods-3793729-supplementary.pdf]

Supplementary Information

Simultaneous detection of four foodborne pathogens in sliced raw freshwater fish based on high-resolution melting curve analysis

Shan Shan[1,2], Xiaoyu Tong[1], Wenyu Du[1], Yin Chen[1], Long Cheng[1], Fang Yan[1,3], Yujie Zhai[1,3], Kui Zhao[2], Haiyan Ni[1], Xiaomei Sha[1], Xiaoqing Liu[2],Chengwei Liu[2], Shuanglong Wang[4], Daofeng Liu[2,*]

[1]College of Life Science, Nanchang Key Laboratory of Microbial Resources Exploitation & Utilization from Poyang Lake Wetland, Jiangxi Normal University, Nanchang 330022, China

[2]Jiangxi Provincial Key Laboratory of Major Epidemics Prevention and Control, Key Laboratory of Nutrition Diet and Health of Jiangxi Provincial Health Commission, Jiangxi Provincial Center for Disease Control and Prevention, 555 East Beijing Road, Nanchang 330029, China

[3]School of Public Health, Jiangxi Medical Collage, Nanchang University, Nanchang 330019, China

[4]Jiangxi Key Laboratory for Mass Spectrometry and Instrumentation, East China University of Technology, Nanchang 330013, China

*Corresponding author: defoelau@163.com

Table S1 List of the strains utilized and their sources

Subspecies name	Abbreviation	Strains Number
Enterobacter sakazakii	*E.sakazakii*	JXCDC-EBS-1
enteroaggregative *Escherichia coli*	EAEC	JXCDC-EAEC-1
enteroinvasive *Escherichia coli*	EIEC	JXCDC-EIEC-1
enteropathogenic *Escherichia coli*	EPEC	JXCDC-EPEC-1
enterotoxigenic *Escherichia coli*	ETEC	JXCDC-ETEC-1
Listeria monocytogenes	LM	CMCC 54009
Listeria monocytogenes	LM	JXCDC-LM-1
Listeria monocytogenes	LM	JXCDC-LM-2
Listeria monocytogenes	LM	JXCDC-LM-3
Listeria monocytogenes	LM	JXCDC-LM-4
Listeria monocytogenes	LM	JXCDC-LM-5
Salmonella Typhimurium	ST	JXCDC-ST-1
Salmonella Typhimurium	ST	JXCDC-ST-2
Salmonella Typhimurium	ST	CMCC 50920
Shiga toxin- producing *Escherichia coli*	STEC	JXCDC-STEC-1
Shigella	*Shigella*	JXCDC-SHI-1
Staphylococcus argenteus	*S.argenteus*	JXCDC-SARG-1
Staphylococcus aureus	SA	ATCC 6583
Staphylococcus aureus	SA	JXCDC-SA-1
Staphylococcus aureus	SA	JXCDC-SA-2
Staphylococcus aureus	SA	JXCDC-SA-3
Vibrio parahaemolyticus	VP	JXCDC-VP-1
Vibrio parahaemolyticus	VP	JXCDC-VP-2
Vibrio parahaemolyticus	VP	JXCDC-VP-3
Vibrio parahaemolyticus	VP	CMCC 20030

Table S2 Primer sequences

Strains	Gene name	Primer name	Primer sequence (5'-3')
Listeria monocytogenes	*hlyA*	*hlyA*-F	CTATTTGCCTGGTAACGC
		hlyA-R	ACCATTCCCAAGCTAAAC
Salmonella Typhimurium	*invA*	*invA*-F	CTCTATTGTCACCGTGGTC
		invA-R	ATCGCACCGTCAAAGGAA
Vibrio parahaemolyticus	*tlh*	*tlh*-F	GTTCGCTGTTGGTATCGC
		tlh-R	AGCCGTCAATGGTGAAGTAG
Staphylococcus aureus	*nuc*	*nuc*-F	GGGCAATACGCAAAGAGG
		nuc-R	GCTTTAATAAATGTCGCAGGTT

Table S3. Theoretical Tm Prediction of Amplification Products for Different Gene Types

**Type**	**Tm (°C)**
hlyA	76.8
tlh	84.3
nuc	79.4
invA	87.8
